# Effect of early postoperative enteral nutrition on the short‐term prognosis in neonatal gastric perforation

**DOI:** 10.1002/pdi3.34

**Published:** 2023-10-18

**Authors:** Ting Zhu, Yu Liu, Huan Wei, Shuo Tang, Xiaowen Li, Mengying Cui, Yuan Shi, Zheng‐Li Wang

**Affiliations:** ^1^ Neonatal Diagnosis and Treatment Center of Children's Hospital of Chongqing Medical University Chongqing China; ^2^ National Clinical Research Center for Child Health and Disorders Chongqing China; ^3^ Ministry of Education Key Laboratory of Child Development and Disorders Chongqing China; ^4^ China International Science and Technology Cooperation Base of Child Development and Critical Disorders Chongqing China; ^5^ National Demonstration Base of Standardized Training Base for Resident Physicians Chongqing China; ^6^ Department of General Surgery & Neonatal Surgery Children's Hospital of Chongqing Medical University Chongqing China

**Keywords:** early enteral nutrition, neonatal gastric perforation, short‐term prognosis

## Abstract

The aim of this retrospective study was to investigate the impact of early postoperative enteral nutrition on the short‐term prognosis of neonatal gastric perforation. The 63 neonates were divided into two groups based on enteral nutrition timing: the early enteral nutrition (EEN) group (≤15 days) and the late enteral nutrition (LEN) group (>15 days). The EEN group was additionally matched with the LEN1 group based on closely aligned gestational age (difference ≤6 days), birth weight (difference <250 g), and age of onset (<1 day). Data from the EEN, LEN, and LEN1 groups were compared and analyzed. No significant differences were observed among the groups in baseline characteristics such as gestational age, birth weight, age at hospital admission, cesarean section rate, and so on (*p* > 0.05). Furthermore, preoperative comorbidities, clinical symptoms, and examination results were not significantly different between the three groups (*p* > 0.05). However, the time required to achieve total enteral nutrition, the length of hospital stay, and fistula retention time were significantly less in the EEN group compared to the LEN groups (*p* < 0.05). The EEN group also exhibited a shorter gastrointestinal decompression time than the LEN1 group, but other major postoperative outcome measures were not significantly different. In conclusion, our study suggests that early postoperative enteral nutrition (≤15 days) could reduce the time to total enteral nutrition, length of hospital stay, and fistula retention time, without increasing adverse prognosis rates.

## Graphical abstract

Our study suggests that early postoperative enteral nutrition (≤15 days) could reduce the time to total enteral nutrition, length of hospital stay, and fistula retention time, without increasing the likelihood of a poor prognosis.

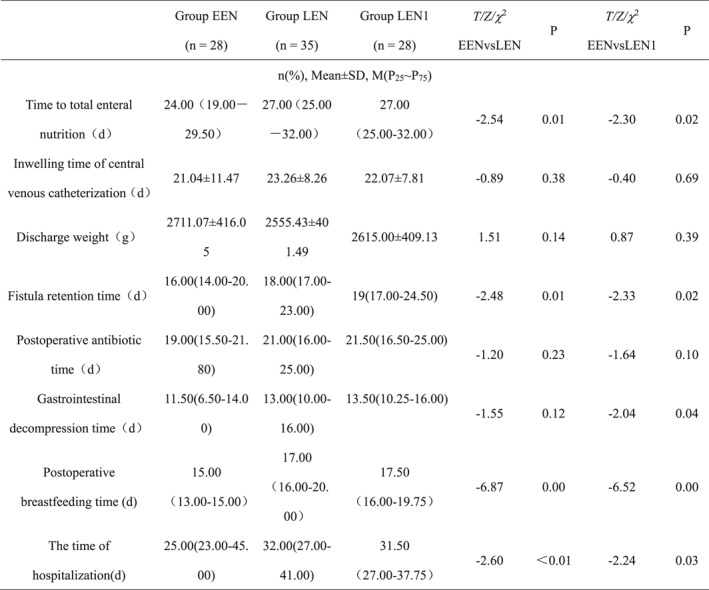

## 1 INTRODUCTION

Neonatal gastric perforation (NGP) is a rare and devastating surgical emergency, accounting for 7% of all neonatal gastrointestinal perforations.^1^, ^2^, ^3^, ^4^ The primary classifications of neonatal gastric perforation are spontaneous gastric perforation and traumatic gastric perforation. The primary risk factors associated with spontaneous NGP include asphyxia, hypoxia, necrotizing enterocolitis (NEC), sepsis, and steroid usage. Traumatic NGPs, on the other hand, generally manifest following gastric tube placement, attempted intubation, or aggressive bag‐mask ventilation.^5^, ^6^, ^7^ A study conducted by Yao Huang et al. reported an alarming mortality rate of 29.7% for NGP. Factors influencing this mortality rate included prematurity, low birth weight, respiratory complications (e.g., bronchopneumonia and hyaline membrane disease), metabolic acidosis (pH <7.3), shock, thrombocytopenia, and sepsis.^8^, ^9^, ^10^

Gastrorrhaphy, both as a standalone procedure and in conjunction with gastrostomy, is the surgical intervention of choice for NGP. The promptness of surgical intervention and the quality of postoperative care play critical roles in infant survival. Nevertheless, surgical site infections (SSIs) following abdominal surgery pose significant clinical challenges. SSIs exhibit various forms, including wound infection, wound dehiscence, anastomotic leakage, postoperative peritonitis, and fistula development. These complications may precipitate prolonged hospital stays, escalated medical costs, diminished quality of life, and elevated mortality rates.^11^, ^12^ In an attempt to mitigate such complications, an extended period of fasting for gastric rest is commonly advised post‐surgery.^13^ This practice, however, predisposes hospitalized patients to malnutrition risks, potentially leading to neurological and gastrointestinal developmental delays and compromised immune function.^14^ Conversely, early microfeeding has been found to modulate immune function and provide protection against infections while simultaneously regulating feeding rate and volume for infants without increasing gastrointestinal strain.^15^ Thus, we hypothesize that early enteral nutrition (EEN) holds significant potential for the intestinal and immune system development in newborns suffering from NGP.

Previous studies involving adults have demonstrated that the initiation of enteral feeding within the first 24 h following gastrointestinal surgery can lead to improved feed tolerance and reduced hospital stay duration.^16^ However, given the heterogeneous nature of NGP pathogenesis, with gastric tissue immaturity often being a primary cause—a factor distinct from adults—the ideal timing for reestablishing feeding in neonates with gastric perforation remains contentious due to a dearth of substantiated, evidence‐based medical studies.^4^, ^17^ The objective of the present study is to assess the effects of early postoperative enteral nutrition on the short‐term prognosis of NGP.

## 2 MATERIALS AND METHODS

**FIGURE 1:**
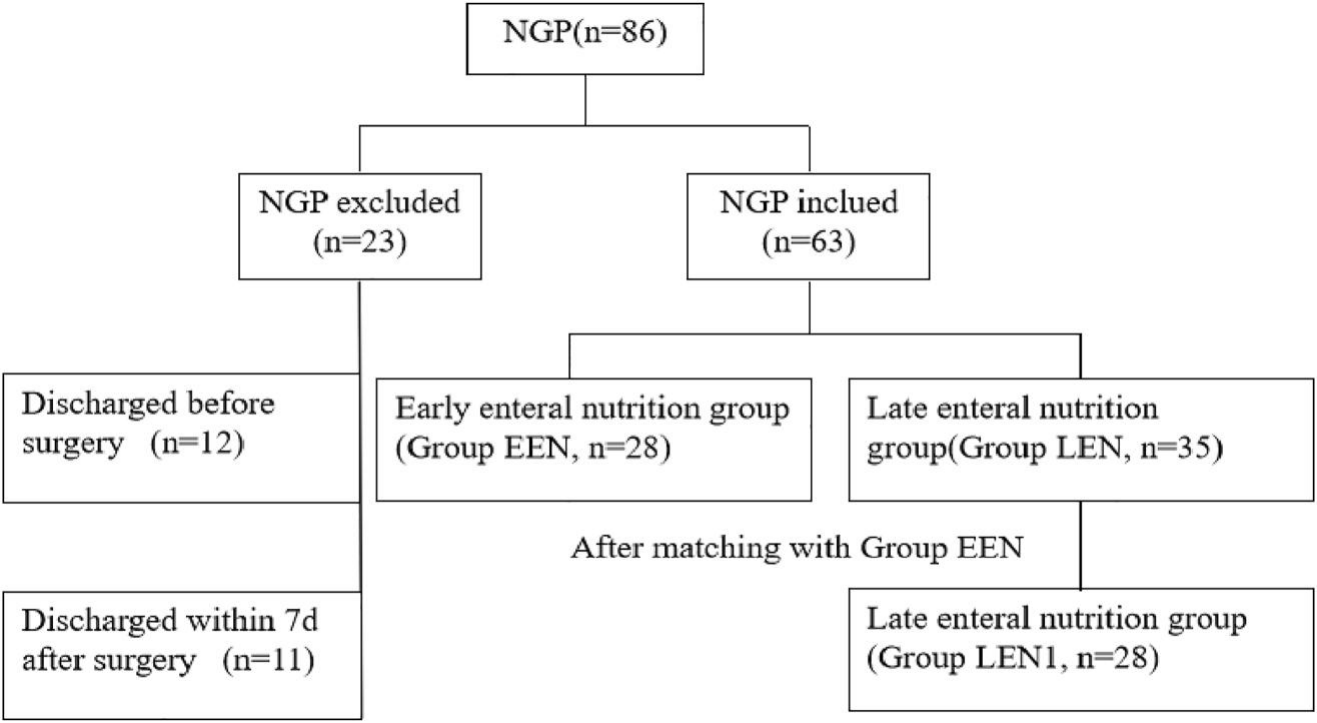
Flow chart for the study population and the subgroups.

Our study involved a retrospective review of medical records pertaining to NGP at the Children's Hospital of Chongqing Medical University between January 2016 and December 2021. A total of 86 neonates with NGP were identified. However, 12 neonates were excluded due to discharge before surgery, and 11 neonates were excluded as they were discharged within 1 week after surgery (Figure 1). The onset of NGP was established as the day when an abdominal X‐ray revealed free gas in the abdominal cavity.^18^ The remaining 63 neonates were divided into two groups: the EEN group, where enteral nutrition was initiated within 15 days post‐surgery (*n* = 28), and the late enteral nutrition (LEN) group, where enteral nutrition was initiated 15 days after surgery (*n* = 35). The EEN group was further matched with a subset of the LEN group (LEN1) based on the differences in birth weight (<250 g), gestational age (<1 week), and age at onset (<1 day). Data from the EEN, LEN, and LEN1 groups were then compared and analyzed. The Institutional Review Board of the Children's Hospital of Chongqing Medical University has approved the study (Approval No. 2022‐387).

### 2.1 Statistical analysis

Statistical analysis was performed using SPSS 21.0 software. Data conforming to normal distribution are expressed as mean ± standard deviation, and comparisons were made using a Student's *t*‐test. Non‐normal distribution data are represented as median and interquartile ranges, and comparisons were made using a nonparametric test. Enumeration data are expressed as percentages, and the *χ* ^2^ test was employed for comparisons between groups. A *p*‐value of <0.05 indicated statistical significance.

## 3 RESULTS

### 3.1 Baseline characteristics of included patients

The present study included a total of 63 infants diagnosed with NGP, 43 (68.3%) of whom were male. As shown in Table 1, there were no significant differences in gestational age (34.46 ± 2.93 weeks vs. 33.88 ± 2.19 weeks vs. 34.09 ± 2.25 weeks) and birth weight (2144.29 ± 516.76 g vs. 2018.57 ± 551.03 g vs. 2148.75 ± 507.41 g) between groups EEN, LEN, and LEN1 (*p* > 0.05). Additionally, other indicators, including gender, maternal age, age of onset, cesarean section rate, pregnancy‐induced hypertension, infants born to diabetic mothers, intrahepatic cholestasis of pregnancy, prenatal antibiotic use, premature rupture of membranes lasting over 18 h, and amniotic fluid fetal staining, did not significantly differ between groups EEN, LEN, and LEN1(*p* > 0.05).

**TABLE 1:** Baseline characteristics of infants with gastric perforation.

	Group EEN (*n* = 28)	Group LEN (*n* = 35)	Group LEN1 (*n* = 28)	***T*/*Z*/*χ* ^2^** **EEN vs. LEN**	*p*	***T*/*Z*/*χ* ^2^** **EEN vs. LEN1**	*p*
	*n* (%), Mean ± SD, *M*(P_25_–P_75_)						
Gender (male)	22 (78.6)	21 (60.0)	17 (60.7)	2.48	0.12	2.11	0.15
Gestational age (w)	34.46 ± 2.93	33.88 ± 2.19	34.09 ± 2.25	0.91	0.37	0.53	0.60
Birth weight (g)	2144.29 ± 516.76	2018.57 ± 551.03	2148.75 ± 507.41	0.93	0.36	−0.03	0.97
Mother's age (y)	30.18 ± 4.23	29.86 ± 5.29	29.32 ± 4.82	0.26	0.80	0.71	0.48
The age at admission (h)	51.50 (2.00–75.25)	36.00 (1.00–86.00)	42.00 (2.00–91.25)	−0.74	0.46	−0.30	0.77
The age onset (d)	2.00 (1.00–3.00)	2.00 (1.50–4.00)	2.00 (1.25–3.00)	−1.31	0.19	−0.69	0.49
Cesarean section	20 (71.4)	32 (91.4)	25 (89.3)	3.04	0.08	2.83	0.09
Pregnancy‐induced hypertension	3 (10.7)	6 (17.1)	5 (17.9)	0.13	0.72	0.15	0.70
Anemia in pregnancy	6 (21.4)	3 (8.6)	3 (10.7)	1.18	0.28	0.53	0.47
Infants of diabetic mother	9 (32.1)	7 (20.0)	6 (21.4)	1.21	0.27	0.82	0.37
Intrahepatic cholestasis of pregnancy	2 (7.1)	1 (2.9)	1 (3.6)	0.04	0.84	0.00	1.00
Prenatal hormone	8 (28.6)	16 (45.7)	13 (46.4)	1.94	0.16	1.91	0.17
Prenatal antibiotics	2 (7.1)	3 (8.6)	3 (10.7)	0.00	1.00	0.00	1.00
Premature rupture of membranes >18 h	4 (14.3)	7 (20.0)	6 (21.4)	0.07	0.80	0.49	0.47
Amniotic fluid fetal staining	3 (10.7)	4 (11.4)	3 (10.7)	0.00	1.00	0.00	1.00
Feeding after birth	16 (57.1)	20 (57.1)	17 (60.7)	0.00	1.00	0.07	0.79

Abbreviations: EEN, early enteral nutrition; LEN, late enteral nutrition; SD, standard deviation.

### 3.2 Preoperative clinical manifestations and comorbidities

As shown in Table 2, no significant differences were found between the two groups, both before and after matching, in preoperative clinical manifestations such as abdominal distension, vomiting, pneumoperitoneum, bloody stool, dyspnea, apnea, fever, normal bowel sounds, red abdominal wall, and visible abdominal wall veins (*p* > 0.05). Likewise, no significant differences (*p* > 0.05) were observed in preoperative comorbidities, including severe pneumonia with respiratory failure, sepsis, asphyxia, congenital heart disease, other digestive tract malformations, and shock between the two groups before and after matching.

**TABLE 2:** Clinical manifestations and comorbidities before surgery.

	Group EEN (*n* = 28)	Group LEN (*n* = 35)	Group LEN1 (*n* = 28)	***χ* ^2^** **EEN vs. LEN**	*p*	***χ* ^2^** **EEN vs. LEN1**	*p*
	*n* (%)						
Severe pneumonia with respiratory failure	19 (67.9)	24 (68.6)	19 (67.9)	0.00	0.95	0.00	1.00
Sepsis	15 (53.6)	19 (54.3)	15 (53.6)	0.00	0.96	0.00	1.00
Asphyxia	5 (17.9)	2 (5.7)	2 (7.1)	1.26	0.26	0.65	0.42
Congenital heart disease	6 (21.4)	10 (28.6)	9 (32.1)	0.42	0.52	0.82	0.37
Other digestive tract malformations	5 (17.9)	5 (14.3)	4 (14.3)	0.00	0.97	0.00	1.00
Shock	5 (17.9)	8 (22.9)	6 (21.4)	0.24	0.63	0.11	0.74
Abdominal distension	27 (96.4)	29 (82.9)	24 (85.7)	1.69	0.19	0.88	0.35
Vomiting	4 (14.3)	8 (22.9)	7 (25)	0.74	0.39	1.02	0.31
Pneumoperitoneum	22 (78.6)	29 (82.9)	22 (78.6)	0.19	0.67	0.00	1.00
Bloody stool	1 (3.6)	1 (2.9)	1 (3.6)		1.00	0.00	1.00
Dyspnea	17 (60.7)	21 (60.0)	17 (60.7)	0.00	0.95	0.00	1.00
Apnea	6 (21.4)	7 (20.0)	5 (17.9)	0.02	0.89	0.11	0.74
Fever	1 (3.6)	0 (0.0)	0 (0.0)		0.44		1.00
Abnormal bowel sounds	23 (82.1)	24 (68.6)	18 (64.3)	1.51	0.22	2.28	0.13
The red abdominal wall	5 (17.9)	6 (17.1)	5 (17.9)	0.00	1.00	0.00	1.00
The abdominal wall vein revealed	8 (28.6)	11 (31.4)	8 (28.6)	0.06	0.81	0.00	1.00

Abbreviations: EEN, early enteral nutrition; LEN, late enteral nutrition.

### 3.3 Laboratory results, treatment of NGP, and post‐surgical comorbidities

As illustrated in Table 3, no significant differences in treatment were observed among the EEN, LEN, and LEN1 groups (*p* > 0.05). Furthermore, there were no marked variances in procalcitonin concentrations or the incidence of SSI among these groups. Other laboratory parameters, including pH, lactic acid, C‐reactive protein, leukocyte count, platelet count, hemoglobin, positive blood culture, and albumin, demonstrated no significant differences among the EEN, LEN, and LEN1 groups (*p* > 0.05).

**TABLE 3:** Laboratory results; treatment of NPG and comorbidities after surgery.

	Group EEN (*n* = 28)	Group LEN (*n* = 35)	Group LEN1 (*n* = 28)	***T/Z/χ* ^2^** **EEN vs. LEN**	*p*	***T/Z/χ* ^2^** **EEN vs. LEN1**	*p*
	*n* (%), Mean ± SD, *M*(P_25_–P_75_)						
Before surgery							
PH	7.34 ± 0.15	7.32 ± 0.12	7.33 ± 0.13	0.61	0.54	0.57	0.57
Lac (mmol/L)	2.10 (1.60–3.30)	2.10 (1.20–2.90)	2.15 (1.13–3.35)	−0.81	0.42	−0.68	0.50
Leukocyte (×10^9^/L)	8.31 (4.73–10.13)	7.93 (3.51–12.99)	10.37 (4.15–13.21)	−0.10	0.92	−0.67	0.50
Platelet count (×10^9^/L)	235.50 (152.00–287.00)	212.00 (146.00–272.00)	232.50 (170.25–288.50)	−0.24	0.81	−0.21	0.83
Hemoglobin (g/L)	150.71 ± 29.47	157.89 ± 28.59	154.68 ± 29.22	−0.98	0.33	−0.45	0.65
PCT (ng/mL)	12.54 (1.28–30.37)	2.10 (0.21–38.56)	1.44 (0.17–23.71)	−1.63	0.10	−1.91	0.51
Elevated CRP	11 (39.3)	14 (40.0)	11 (39.3)	0.00	0.95	0.00	1.00
Positive blood culture	1 (3.6)	1 (2.9)	0 (0)		1.00		1.00
Blood sodium (mmol/L)	136.39 ± 4.48	136.71 ± 4.21	137.01 ± 4.05	−0.29	0.77	−0.54	0.59
Albumin (g/L)	26.00 (24.03–28.80)	27.50 (24.10–31.00)	26.80 (24.10–30.45)	−0.92	0.36	−0.38	0.71
After surgery							
Albumin (g/L)	27.26 ± 6.46	26.51 ± 6.18	26.68 ± 6.50	0.47	0.64	0.34	0.74
Blood sodium (mmol/L)	140.00 (137.33–142.98)	140.00 (136.00–143.00)	140.12 ± 4.78	−0.48	0.63	0.04	0.97
Edema	18 (64.3)	29 (82.9)	23 (82.1)	2.83	0.09	2.28	0.13
Sepsis	20 (71.4)	28 (80.0)	23 (82.1)	0.63	0.43	0.90	0.34
Intracranial hemorrhage	6 (21.4)	11 (31.4)	9 (32.1)	0.79	0.37	0.82	0.37
Coagulopathy	20 (71.4)	26 (74.3)	20 (71.4)	0.06	0.80	0.00	1.00
Preoperative antibiotics	25 (89.3)	30 (85.7)	25 (89.3)	0.00	0.97	0.00	1.00
Gastrostomy	24 (85.7)	33 (94.3)	26 (92.9)	0.52	0.47	0.19	0.67
Abdominal drainage	24 (85.7)	25 (71.4)	19 (67.9)	1.84	0.18	2.50	0.11
Ascites (mL)	100.00 (50.00–150.00)	100.00 (50.00–150.00)	125.00 (50.00–150.00)	−0.27	0.78	−0.62	0.53
Gastric perforation length (cm)	5.00 (4.00–6.75)	5.00 (4.00–6.00)	5 (3.25–6.00)	1.21	0.23	−1.28	0.20
Age of surgery (d)	2.00 (2.00–3.75)	3.00 (2.00–5.00)	3.00 (2.00–4.00)	−1.79	0.07	1.24	0.21
The operative times (h)	2.46 ± 1.14	2.26 ± 0.78	2.32 ± 0.95	0.75	0.45	0.51	0.61
Hepatic dysfunction	3 (10.7)	2 (5.7)	2 (7.1)	0.07	0.79	0.00	1.00
Renal dysfunction	9 (32.1)	16 (45.7)	14 (50.0)	1.20	0.27	1.85	0.17
SSI	3 (10.7)	9 (25.7)	7 (10.7)	2.27	0.13	1.00	0.16

Abbreviations: CRP, C‐reactive potein; EEN, early enteral nutrition; LEN, late enteral nutrition; NGP, neonatal gastric perforation; PCT, procalcitonin; PH, power of hydrogen; SD, standard deviation; SSI, surgical site infection.

Moreover, no significant differences were observed among the three groups in comorbidities, including edema, sepsis, intracranial hemorrhage, coagulopathy, hepatic dysfunction, renal dysfunction, albumin levels, and blood sodium levels (*p* > 0.05).

### 3.4 Effect of EEN on the prognosis of NGP

Table 4 highlights significant differences in the time required to achieve total enteral nutrition for both matched and unmatched groups (*p* < 0.05). The EEN group reached total enteral nutrition more quickly than the other two groups. The length of hospital stay(LOS) for the EEN and LEN groups was 25 (23–45) days and 32 (27–41) days, respectively, the shorter hospitalization duration was associated with EEN (*p* < 0.05). Additionally, the gastrointestinal decompression time in the EEN group was shorter than that in the LEN1 group (*p* < 0.05). The fistula tube indwelling time was also shorter in the EEN group when compared to the LEN and LEN1 groups (*p* = 0.01 and *p* = 0.02).

**TABLE 4:** The effect of EEN on the prognosis of NGP.

	Group EEN (*n* = 28)	Group LEN (*n* = 35)	Group LEN1 (*n* = 28)	***T/Z/χ* ^2^** **EEN vs. LEN**	*p*	***T/Z/χ* ^2^** **EEN vs. LEN1**	*p*
	*n* (%), Mean ± SD, *M*(P_25_–P_75_)						
Time to total enteral nutrition (d)	24.00 (19.00–29.50)	27.00 (25.00–32.00)	27.00 (25.00–32.00)	−2.54	0.01	−2.30	0.02
Inwelling time of central venous catheterization (d)	21.04 ± 11.47	23.26 ± 8.26	22.07 ± 7.81	−0.89	0.38	−0.40	0.69
Discharge weight (g)	2711.07 ± 416.05	2555.43 ± 401.49	2615.00 ± 409.13	1.51	0.14	0.87	0.39
Fistula retention time (d)	16.00 (14.00–20.00)	18.00 (17.00–23.00)	19 (17.00–24.50)	−2.48	0.01	−2.33	0.02
Postoperative antibiotic time (d)	19.00 (15.50–21.80)	21.00 (16.00–25.00)	21.50 (16.50–25.00)	−1.20	0.23	−1.64	0.10
Gastrointestinal decompression time (d)	11.50 (6.50–14.00)	13.00 (10.00–16.00)	13.50 (10.25–16.00)	−1.55	0.12	−2.04	0.04
Postoperative invasive respiratory support time (d)	3.00 (2.00–4.00)	3.00 (2.00–5.00)	3.00 (2.00–5.00)	−0.27	0.79	−0.33	0.74
Postoperative plasma infusion frequency (f)	1.00 (0.00–2.00)	1.00 (0.00–2.00)	1.00 (0.00–2.00)	−0.87	0.38	−0.18	0.86
Postoperative albumin infusion dose (g/kg)	11.50 (8.00–18.25)	11.00 (7.00–15.00)	10.50 (7.00–15.00)	−0.23	0.82	−0.47	0.64
Secondary surgery	2 (7.1)	3 (8.6)	3 (10.7)	0.00	1.00	0.00	1.00
Postoperative initiation time of feeds (d)	15.00 (13.00–15.00)	17.00 (16.00–20.00)	17.50 (16.00–19.75)	−6.87	0.00	−6.52	0.00
The time of hospitalization (d)	25.00 (23.00–45.00)	32.00 (27.00–41.00)	31.50 (27.00–37.75)	−2.60	<0.01	−2.24	0.03

Abbreviations: EEN, early enteral nutrition; LEN, late enteral nutrition; NGP, neonatal gastric perforation; SD, standard deviation.

However, no significant differences were observed between the matched and unmatched groups concerning peripherally inserted central venous catheter indwelling time, discharge weight, frequency of postoperative plasma infusion, duration of postoperative antibiotic use, postoperative invasive respiratory support time, postoperative breastfeeding time, and postoperative albumin infusion dose.

## 4 DISCUSSION

In this study, we examined 63 neonates who underwent surgery for gastric perforation, focusing on their general condition, clinical manifestations, surgical factors, lactation timing, and postoperative complications. We found that the time to achieve total enteral nutrition, LOS, and fistula tube indwelling duration were significantly shorter in the EEN group compared to the LEN group (*p* < 0.05). Nevertheless, we observed no significant differences in postoperative outcomes or short‐term prognosis between the groups.

The precise etiology of NGP remains elusive, with potential specific causes including congenital agenesis of the gastric musculature, gastric wall ischemia arising from asphyxia, and elevated intragastric pressure due to C‐KIT mast cell deficiency.^19^, ^20^ Recent studies have also implicated gastrointestinal anomalies such as diaphragmatic hernia, NEC, duodenal atresia, gastroschisis, and omphalocele as potential risk factors for mechanical gastric perforations.^21^, ^22^ The median time of initiation of feeds in the early nutrition group was 7 days for surgically treated NEC.^23^ Conversely, the median initiation time for EEN was observed to be 15 days. This discrepancy could be attributed to congenital absence of the gastric muscle and elevated gastric acidity, both of which obstruct stomach repair and necessitate an extended fasting period compared to other abdominal surgeries.

At present, there is no established consensus regarding the optimal timing of enteral nutrition following NGP surgery. Previous research has revealed that early microfeeding stimulates intestinal epithelial cells and gastrointestinal hormones, thereby bolstering the intestinal defense mechanism, priming the gut for substantial feeding, and diminishing the duration to attain sufficient nutrition.^15^, ^24^ Conversely, protracted fasting can result in intestinal mucosal villi atrophy, reduction in gastrointestinal blood flow, onset of digestive dysfunction, and delayed development of the mucosal epithelium and innate immune function.^25^, ^26^ Nonetheless, Hayashi et al. have asserted that early jejunal nutrition may instigate feeding complications, compelling infants to curtail or cease enteral nutrition intake and consequently prolonging the time to attain total enteral nutrition.^27^ Our research corroborates that EEN can expedite the attainment of adequate nutrition, aligning with prior studies.^28^ Therefore, pediatricians might consider initiating enteral feeding early, while simultaneously monitoring the patient's tolerance to enteral nutrition.

SSIs following abdominal surgery represent a significant and frequently encountered clinical challenge, which often dissuades pediatricians from initiating EEN. However, our study revealed no association between the duration of fasting and the incidence of SSIs. Immediate postoperative enteral feeding has demonstrated efficacy in diminishing septic morbidity in high‐risk surgical patients.^29^ There is a recognized association between prolonged fasting, extended venous catheterization, and an elevated probability of opportunistic infections and sepsis. Numerous studies have corroborated this positive correlation between catheter duration and sepsis risk.^30^, ^31^ Patients receiving EEN also exhibited a trend toward a lower sepsis incidence, though the difference was not statistically significant. This outcome may be attributable to enhanced management of peripherally inserted central venous catheters and the small sample size in our study. Additionally, extended exposure to parenteral nutrition and sustained enteral starvation are recognized risk factors for cholestasis.^32^, ^33^ Our results corroborate that early EEN can curtail the duration of intravenous nutrition without exacerbating other complications.

Our study found a marked distinction in the duration of gastrointestinal decompression in relation to gestational age and birth weight, a compelling observation that suggests a clear influence of these parameters on decompression times.

Despite considerable strides in neonatology, NGP continues to present as a seldom occurring but potentially lethal surgical emergency. Even after surviving this condition, infants may face diverse health challenges, such as developmental delay, iron‐deficiency anemia, and steatorrhea (fatty stools), underscoring the severity of the disease's aftermath. Previous research has established the viability of EEN in pediatric patients following intestinal surgery.^34^, ^35^ Our investigation supports these findings, providing evidence that early initiation of enteral feeding can significantly reduce the duration of intravenous nutrition, without exacerbating other complications. An inverse correlation was discovered between the timing of enteral feeding initiation and the length of hospital stay. The expeditious initiation of enteral feeding correlated significantly with a reduced hospitalization period, thereby potentially decreasing healthcare expenses, and perhaps offering a neurodevelopmental advantage due to earlier discharge and enhanced familial interaction.^13^ In instances where newborns cannot be promptly discharged due to other medical complications, kangaroo care can be employed to bolster the development of the nervous system.^36^

Contrary to initial predictions, our findings revealed that the disparity in discharge weight between EEN and LEN groups was statistically insignificant. With the administration of parenteral nutrition, we found that caloric intake was sufficient for growth, leading to negligible variations in discharge weight between the two groups.

We acknowledge several limitations in our study, including the sample size and the inherent errors and biases associated with retrospective studies. Therefore, it is inevitable to expand the sample size and conduct a multicenter study with a larger population in order to provide more conclusive evidence. Although our findings are compelling, they should be interpreted with caution in the absence of large randomized controlled trials. A prospective randomized study may be necessary to further substantiate our conclusions.

## 5 CONCLUSION

Our study suggests that the application of early postoperative enteral nutrition (≤15 days) can significantly reduce the time required to achieve total enteral nutrition, shorten hospital stay duration, and decrease fistula retention time without increasing adverse prognosis rates. Hence, it is recommended that pediatric practitioners incorporate EEN strategies when managing newborns after gastric perforation surgery.

## AUTHOR CONTRIBUTIONS

Ting Zhu and Zheng‐Li Wang designed this study, Ting Zhu contributed to the overall drafting of the manuscript, Yu Liu and Huan Wei led the data extraction, Shuo Tang and Mengying Cui led the statistical analysis, Xiaowen Li, Yuan Shi, and Zheng‐Li Wang contributed to the paper revision. All authors have approved the publication of this protocol.

## CONFLICT OF INTEREST STATEMENT

There are no competing interests to declare.

## ETHICS STATEMENT

There is no necessity for this study to acquire ethical approval, since no private information of participants will be involved. Results of the present study will be disseminated in a peer‐reviewed journal or conference presentation.

## Data Availability

Data are available upon reasonable request. All data relevant to the study are included in the article. The data that support the findings of this study are available upon reasonable request from the corresponding author.
